# Association between physical activity and episodic memory and the moderating effects of the apolipoprotein E ε4 allele and age

**DOI:** 10.3389/fnagi.2023.1184609

**Published:** 2023-07-11

**Authors:** Boung Chul Lee, Young Min Choe, Guk-Hee Suh, Ihn-Geun Choi, Hyun Soo Kim, Jaeuk Hwang, Dahyun Yi, Jee Wook Kim

**Affiliations:** ^1^Department of Psychiatry, Hallym University College of Medicine, Chuncheon, Republic of Korea; ^2^Department of Neuropsychiatry, Hallym University Hangang Sacred Heart Hospital, Seoul, Republic of Korea; ^3^Department of Neuropsychiatry, Hallym University Dongtan Sacred Heart Hospital, Gyeonggi, Republic of Korea; ^4^Department of Psychiatry, Seoul W Psychiatric Office, Seoul, Republic of Korea; ^5^Department of Laboratory Medicine, Hallym University Dongtan Sacred Heart Hospital, Gyeonggi, Republic of Korea; ^6^Department of Psychiatry, Soonchunhyang University Hospital Seoul, Seoul, Republic of Korea; ^7^Institute of Human Behavioral Medicine, Medical Research Center Seoul National University, Seoul, Republic of Korea

**Keywords:** physical activity, memory, Alzheimer’s disease, age, APOE4

## Abstract

**Background:**

An abundance of evidence indicates that physical activity may protect against Alzheimer’s disease (AD) and related cognitive decline. However, little is known about the association between physical activity and AD-related cognitive decline according to age and the apolipoprotein E (APOE) ε4 allele (APOE4) as major risk factors. Therefore, we examined whether age and APOE4 status modulate the effects of physical activity on episodic memory as AD-related cognition in non-demented older adults.

**Methods:**

We enrolled 196 adults aged between 65 and 90 years, with no dementia. All participants underwent comprehensive clinical assessments including physical activity evaluation and APOE genotyping. The AD-related cognitive domain was assessed by the episodic memory, as the earliest cognitive change in AD, and non-memory cognition for comparative purposes. Overall cognition was assessed by the total score (TS) of the Consortium to Establish a Registry for Alzheimer’s Disease neuropsychological battery.

**Results:**

We found significant physical activity × age and physical activity × APOE4 interaction effects on episodic memory. Subgroup analyses indicated that an association between physical activity and increased episodic memory was apparent only in subjects aged > 70 years, and in APOE4-positive subjects.

**Conclusion:**

Our findings suggest that physical activity has beneficial effects on episodic memory, as an AD-related cognitive domain, in individuals aged > 70 years and in APOE4-positive individuals. Physicians should take age and APOE4 status account into when recommending physical activity to prevent AD-related cognitive decline.

## Key points

### Question

• Is physical activity associated with reduced Alzheimer’s disease (AD)-related cognitive dysfunction in humans? Is such an effect influenced by age or apolipoprotein E (APOE) ε4 allele (APOE4) status, which are the strongest risk factors for AD?

### Findings

• In this study of 196 non-demented older adults, physical activity was positively associated with episodic memory, as the earliest cognitive change in AD, but not with non-memory cognition. The benefits of physical activity on episodic memory were apparent only in older adults > 70 years and in APOE4-positive individuals.

### Meaning

• Physical activity in non-demented adults had a beneficial effect on episodic memory, as an AD-related cognitive domain, in individuals aged > 70 years and in APOE4-positive individuals. Physicians should take age and APOE4 status account into when recommending physical activity to prevent AD-related cognitive decline.

## Introduction

Alzheimer’s disease (AD) is the most common progressive neurodegenerative disease among old adults. It is characterized by two core pathologies: extracellular aggregates of beta-amyloid peptide (Aβ) and intracellular aggregates of hyperphosphorylated tau proteins (Hardy and Selkoe, 2002). Due to the lack of effective drugs, promising evidence-based modifiable lifestyle approaches such as physical activity are emerging as alternatives to protection for AD. Accumulating evidence suggests a protective link between physical activity and AD or related cognitive dysfunction (Albert et al., 1995; Lautenschlager et al., 2008; Leyland et al., 2019; Ruiz-Muelle and López-Rodríguez, 2019; Jeon et al., 2020; Abasıyanık et al., 2021; Roeh et al., 2021; Sohn et al., 2022).

Age (Evans et al., 1989; Murman, 2015) and apolipoprotein E (APOE) ε4 allele (APOE4) (Yamazaki et al., 2019) are the strongest risk factors for biological and genetic AD, respectively. Physical activity may yield cognitive improvements (Colcombe and Kramer, 2003; Kramer and Colcombe, 2018) and lower dementia incidence (Heyn et al., 2004; Larson et al., 2006) in older adults without dementia. However, little is known about the association between physical activity and episodic memory decline, which is the earliest sign of AD-related cognitive change according to age and APOE4 as major risk factors for AD-related cognitive decline. Therefore, we examined whether age and APOE4 status modulate the effects of physical activity on episodic memory as AD-related cognition in non-demented older adults, as well as the protective effects of physical activity on episodic memory.

## Materials and methods

### Participants

This study was part of the General Lifestyle and AD (GLAD) study, an ongoing prospective cohort study that began in 2020 (Choe et al., 2022). As of September 2022, 196 non-demented adults aged 65–90 years were enrolled, including 113 cognitively normal (CN) adults and 83 with mild cognitive impairment (MCI). Participants were recruited among older adults who had attended a dementia screening program at the memory clinic of Hallym University Dongtan Sacred Heart Hospital, Hwaseong, Republic of Korea (Choe et al., 2022). Volunteers were invited for an eligibility assessment. Additional volunteers were recruited from the community through recommendations from other participants, family members, friends, or acquaintances. The CN group consisted of participants with a Clinical Dementia Rating (Morris, 1993) score of 0 and no diagnosis of MCI or dementia. All participants with MCI met the current consensus criteria for amnestic MCI, including memory complaints confirmed by an informant, objective memory impairment, preservation of global cognitive function, independence in functional activities, and the absence of dementia (Petersen, 2004). In the objective memory impairment assessment, the age-, education-, and sex-adjusted z-score was < –1.0 on at least one of four episodic memory tests in the Korean version of the Consortium to Establish a Registry for Alzheimer’s Disease (CERAD) neuropsychological battery: (Morris et al., 1989; Lee et al., 2002) word list memory, word list recall, word list recognition, and the constructional recall test (Morris et al., 1989; Lee et al., 2002, 2004; Choe et al., 2022). All individuals with MCI had a Clinical Dementia Rating score of 0.5 (Petersen, 2004). The exclusion criteria were major psychiatric illness, a significant neurological or medical condition or comorbidity that could affect mental functioning, illiteracy, visual/hearing difficulty, severe communication or behavioral problems that would make clinical examinations difficult, and the use of an investigational drug (Choe et al., 2022). The study protocol was approved by the Institutional Review Board of the Hallym University Dongtan Sacred Heart Hospital and the study was conducted it in accordance with the recommendations of the current version of the Declaration of Helsinki (Choe et al., 2022). The participants or their legal representatives provided informed consent.

### Clinical assessments

All participants underwent standardized clinical assessments by trained psychiatrists, which incorporates the CERAD clinical and neuropsychological battery (Morris et al., 1989; Lee et al., 2002). Trained neuropsychologists administered the neuropsychological battery (Lee et al., 2004) to all participants. AD-related cognitive domain was measured in terms of the episodic memory, as the earliest cognitive change in AD, (Howieson et al., 1997; Grober et al., 2000; Laakso et al., 2000; Bäckman et al., 2001, 2005; Tromp et al., 2015), and non-memory cognition for comparative purposes. The episodic memory score was determined by summing the scores of four episodic memory tests (word list memory, word list recall, word list recognition, and constructional recall) in the CERAD neuropsychological battery. The non-memory score was calculated by summing the scores of three non-memory tests (verbal fluency, modified Boston naming test, and constructional praxis) in the CERAD neuropsychological battery. Overall cognition was also assessed in terms of the total score (TS) of the Consortium to Establish a Registry for Alzheimer’s Disease neuropsychological battery. TS was generated by summing the scores of seven tests in the CERAD neuropsychological battery (verbal fluency, modified Boston naming, word list memory, constructional praxis, word list recall, word list recognition, and constructional recall) (Seo et al., 2010).

We used the Geriatric Depression Scale (GDS) to measure the severity of depressive symptoms (Yesavage et al., 1982; Kim et al., 2008). Vascular risks such as hypertension, diabetes mellitus, dyslipidemia, coronary heart disease, transient ischemic attack, and stroke were assessed based on data collected by trained researchers during systematic interviews of the participants and their families. The vascular risk score (VRS) was calculated based on the number of vascular risk factors present and was reported as a percentage (DeCarli et al., 2004). Alcohol intake status (never/former/drinker) was also evaluated through trained researcher interviews and a medical record review. Dietary patterns (i.e., consumption of proteins, vegetables, fruits, salty foods, fatty foods, and fried foods; meal frequency; and inter-meal snacking) were systematically assessed using the mini-dietary assessment (Kim et al., 2003), which is a brief, valid nutritional evaluation tool for Korean populations (Jang et al., 2008). The accuracy of the data was ensured by interviewing reliable informants.

### Assessment of physical activity

Physical activity was evaluated using the Korean-version of the Physical Activity Scale for the Elderly (PASE) (Washburn et al., 1993; Choe et al., 2010), which has sufficient reliability and validity. Trained researchers assessed the frequency, duration, and intensity of participants’ leisure, household, and occupational activities during the previous week. The test items were weighted and a PASE score was obtained by summing the PASE subscale scores for leisure, household, and occupational activities. A higher score indicated greater physical activity. Participants were divided into low (used as a reference category), medium, and high categories based on PASE score using tertiles.

### Assessment of motor signs

Gait scores were derived based on the motor subscale of the Unified Parkinson’s Disease Rating Scale (UPDRS) (Movement Disorder Society Task Force on Rating Scales for Parkinson’s Disease, 2003) to determine the impact of physical activity on AD-related cognitive domains by excluding gait instability, which could be affected by preexisting brain diseases. Gait accounts for a significant proportion of physical activity because it is a complex motor movement that requires the coordination of all body parts (Park et al., 2012).

### Blood test

After overnight fasting, blood samples were obtained from the subjects by venipuncture in the morning (8:00–9:00). Hemoglobin levels were determined using an automated hematologic analyzer (XN-3000; Sysmex, Kobe, Japan) and dedicated reagents. Albumin and glucose levels were measured using an analyzer (COBAS c702; Roche Diagnostics, Mannheim, Germany) and dedicated reagents. Copper and zinc levels were measured using an inductively coupled plasma-mass spectrometer (ELAN DRC-e; Perkin Elmer, Waltham, MA, USA).

### APOE4 genotyping

Blood samples were collected in EDTA anticoagulated vacutainer tube. DNA was extracted using QIAamp DSP DNA Blood mini kit (QIAGEN, Hilden, Germany) and QIAcube HT System (QIAGEN, Hilden, Germany). APOE genotyping was performed using a Seeplex ApoE ACE Genotyping Kit (Seegene, Seoul, Republic of Korea) and ProFlex PCR system (ThermoFisher Scientific, Waltham, MA, USA) according to the manufacturer’s instruction. PCR product was analyzed using a capillary electrophoresis device (QIAxcel Advanced System, QIAGEN, Hilden, Germany), and interpreted as ε2/ε2, ε2/ε3, ε2/ε4, ε3/ε3, ε3/ε4, or ε4/ε4 according to the electrophoresis pattern and manufacturer’s instruction. APOE4-positive was defined as the presence of at least one ε4 allele.

### Statistical analyses

To examine the relationship between physical activity (continuous and categorical variables) and cognition (TS, memory score, and non-memory score), multiple linear regression analyses were performed with physical activity as the independent variable and cognition as the dependent variable. Because various factors may influence the association between physical activity and cognition, we systematically evaluated all participants to identify potential confounders such as age, sex, APOE4 status, education, clinical diagnosis, depression, vascular risks, alcohol intake, dietary patterns, and blood nutritional markers. We tested two models, adjusting for the covariates in a stepwise manner. The first model included age, sex, APOE4 status, education, clinical diagnosis, depression, alcohol intake, vascular risks, and dietary patterns as covariates; the second model included these covariates plus including blood nutritional markers.

The moderating effects of covariates such as age, sex, APOE4 status, education, clinical diagnosis, GDS score, VRS, and alcohol intake on the association between physical activity and cognition was examined using multiple linear regression analyses including two-way interaction terms for the association between physical activity and cognition as additional independent variables. When an interaction was determined, linear regression analyses were repeated individually depending on the covariate. All statistical analyses were performed using the SPSS Statistics v28 software (IBM Corp, Armonk, NY, USA).

## Results

### Participant characteristics

Table 1 presents the demographic and clinical characteristics of the participants. All participants were physically capable (i.e., UPDRS gait score ≤2). Among the 196 participants, 65, 66, and 66 participants were categorized into the low, medium, and high physical activity groups, respectively.

**TABLE 1:** Baseline participant characteristics according to PASE categories.

Characteristic	PASE				
	**Total**	**Low**	**Medium**	**High**	***p***
*n*	196	65	66	65	
Age, y	72.65 (5.95)	73.76 (6.57)	72.77 (5.53)	71.18 (5.46)	0.051^a^
Female, No. (%)	138 (70.41)	42 (64.62)	53 (80.30)	43 (66.15)	0.095^b^
Education, No. (%)					0.351^b^
≥13 year	42 (21.43)	16 (24.62)	14 (21.21)	12 (18.46)	
10–12 year	51 (26.02)	16 (24.62)	16 (24.24)	19 (29.23)	
4–9 year	85 (43.37)	28 (43.08)	33 (50.00)	24 (43.08)	
0–3 year	18 (9.18)	5 (7.69)	3 (4.55)	10 (15.38)	
MMSE	25.58 (3.45)	25.38 (3.75)	25.34 (3.59)	26.12 (2.85)	0.373^a^
APOE4 positivity, No. (%)	42 (21.43)	12 (18.46)	15 (22.73)	15 (23.08)	0.775^b^
Clinical diagnosis, CN, No. (%)	113 (57.65)	36 (55.38)	35 (53.03)	42 (64.62)	0.367^b^
VRS, %	23.98 (18.58)	26.23 (18.51)	24.18 (18.85)	21.05 (18.24)	0.300^a^
GDS score	10.92 (7.24)	10.84 (7.11)	11.38 (7.23)	10.44 (7.51)	0.763^a^
UPDRS, Gait disturbance requiring assistance	0 (0.00)	0 (0.00)	0 (0.00)	0 (0.00)	
Alcohol drink status, No (%)					0.179^b^
Never	107 (54.59)	30 (46.15)	41 (62.12)	36 (55.38)	
Former	34 (17.35)	16 (24.62)	9 (13.64)	12 (18.46)	
Drinker	55 (28.06)	19 (29.23)	19 (28.79)	17 (26.15)	
**Blood markers**					
Hemoglobin	13.33 (1.51)	13.17 (1.72)	13.33 (1.48)	13.51 (1.26)	0.459^a^
Albumin	4.57 (0.26)	4.56 (0.27)	4.59 (0.26)	4.56 (0.25)	0.736^a^
Glucose, fasting	108.15 (19.94)	107.76 (15.62)	107.23 (17.49)	109.79 (26.64)	0.760^a^
zinc	80.77 (11.15)	80.39 (9.87)	79.99 (11.92)	82.21 (11.66)	0.507^a^
Copper	94.26 (16.73)	93.70 (15.22)	95.27 (18.47)	93.64 (16.34)	0.817^a^
**Nutritional markers**					
Protein, No (%)					0.047^b^
High (Always)	44 (22.45)	19 (29.23)	15 (22.73)	10 (15.38)	
Moderate (Sometimes)	81 (41.33)	18 (27.62)	33 (50.00)	30 (46.15)	
Low (Seldom)	71 (36.22)	28 (43.08)	18 (27.27)	25 (38.46)	
Fruit, No (%)					
High (Always)	109 (55.61)	34 (52.31)	41 (62.12)	34 (51.52)	0.772^b^
Moderate (Sometimes)	44 (22.45)	15 (23.08)	13 (19.70)	16 (24.62)	
Low (Seldom)	43 (21.94)	16 (24.62)	12 (18.18)	15 (1.54)	
Vegetables, No (%)					0.123^b^
High (Always)	125 (63.78)	39 (60.00)	45 (68.18)	41 (63.08)	
Moderate (Sometimes)	45 (22.96)	14 (21.54)	11 (16.67)	20 (30.77)	
Low (Seldom)	26 (13.27)	12 (18.46)	10 (15.15)	4 (6.15)	
Fried foods, No (%)					0.363^b^
High (Always)	21 (10.71)	6 (9.23)	6 (9.09)	9 (13.85)	
Moderate (Sometimes)	24 (12.24)	12 (18.46)	7 (10.60)	5 (7.69)	
Low (Seldom)	148 (75.51)	47 (72.31)	52 (78.79)	49 (75.38)	
Fatty foods, No (%)					0.858^c^
High (Always)	13 (6.63)	6 (9.23)	4 (6.06)	3 (4.62)	
Moderate (Sometimes)	27 (13.78)	9 (13.85)	8 (12.12)	10 (15.38)	
Low (Seldom)	154 (78.57)	50 (76.92)	53 (80.30)	51 (78.46)	
Salty foods, No (%)					0.022^b^
High (Always)	23 (11.73)	6 (9.23)	5 (7.58)	12 (18.46)	
Moderate (Sometimes)	27 (13.78)	15 (23.08)	8 (12.12)	4 (6.15)	
Low (Seldom)	144 (73.47)	44 (67.69)	52 (78.79)	48 (73.85)	
Three meals, No (%)					0.180^b^
High (Always)	114 (58.16)	46 (70.77)	33 (50.00)	35 (53.85)	
Moderate (Sometimes)	64 (32.65)	15 (23.08)	25 (37.88)	24 (36.92)	
Low (Seldom)	16 (8.16)	4 (6.15)	7 (10.61)	5 (7.69)	
Inter-meal snack, No (%)					0.141^b^
High (Always)	114 (58.16)	48 (73.85)	35 (53.03)	31 (47.69)	
Moderate (Sometimes)	64 (32.65)	15 (23.08)	28 (34.85)	21 (32.30)	
Low (Seldom)	16 (8.16)	5 (7.69)	7 (10.61)	4 (6.15)	
**CERAD cognition**					
Total score	69.98 (15.61)	68.12 (17.79)	69.58 (14.52)	72.70 (13.96)	0.254^a^
Episodic memory score	35.10 (9.48)	34.53 (7.31)	34.52 (9.70)	37.05 (9.48)	0.174^a^
Non-memory score	34.25 (6.62)	33.53 (7.31)	34.49 (6.05)	34.81 (6.45)	0.523^a^

PASE, physical activity scale for the elderly; MMSE, mini-mental status examination; APOE4, apolipoprotein ε4; CN, cognitively normal; MCL, minimum cost of living; VRS, vascular risk score; GDS, geriatric depression scale; UPDRS, Unified Parkinson’s Disease Rating Scale; CERAD, Consortium to Establish a Registry for Alzheimer’s Disease.

Data are expressed as mean (standard deviation), unless otherwise indicated.

^a^ by one-way analysis of variance.

^b^ by chi-square test.

^c^ by fisher exact test.

### Association between physical activity and cognition

**FIGURE 1:**
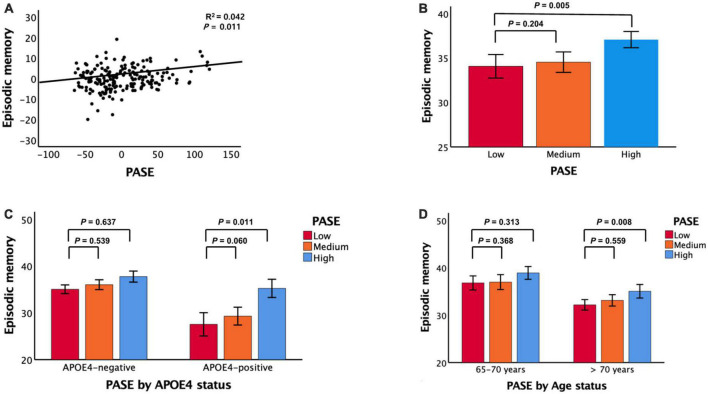
Associations between PASE and episodic memory. **(A)** PASE vs. episodic memory, **(B)** PASE categories vs. episodic memory, **(C)** PASE categories vs. episodic memory according APOE4 status, and **(D)** PASE categories vs. episodic memory according to age. PASE physical activity scale for the elderly, APOE4 apolipoprotein ε4. In panels **(A–D)**, data are adjusted for all potential covariates. In panels **(B–D)**, data are mean cognition values and error bars represent standard error.

After controlling for all confounding factors, the PASE score was positively associated with the episodic memory score but not the non-memory score (Figure 1 and Table 2). It was also positively associated with TS.

**TABLE 2:** Results of multiple linear regression analyses for the associations between PASE and cognition.

	Total score		Episodic memory score		Non-memory score	
	**β**	***p***	**β**	***p***	**β**	***p***
**Overall**						
**Model 1** ^a^						
PASE	0.103	0.042	0.109	0.017	0.060	0.324
**Model 2** ^b^						
PASE	0.107	0.039	0.118	0.011	0.060	0.340
**APOE4-negative**						
**Model 1** ^a^						
PASE	0.016	0.940	0.127	0.486	−0.284	0.214
**Model 2** ^b^						
PASE	0.098	0.653	0.122	0.545	−0.234	0.377
**APOE4-positive**						
**Model 1** ^a^						
PASE	0.130	0.019	0.115	0.028	0.099	0.140
**Model 2** ^b^						
PASE	0.132	0.020	0.123	0.021	0.104	0.139
**Young-old age (65–70 years)**						
**Model 1** ^a^						
PASE	0.056	0.475	0.045	0.547	−0.025	0.809
**Model 2** ^b^						
PASE	0.115	0.185	0.063	0.449	0.040	0.729
**Old-old age (> 70 years)**						
**Model 1** ^a^						
PASE	0.022	0.105	0.155	0.017	0.073	0.387
**Model 2** ^b^						
PASE	0.099	0.182	0.147	0.025	0.062	0.482

PASE, physical activity scale for the elderly; APOE4, apolipoprotein ε4; GDS, geriatric depression scale; VRS, vascular risk score.

^a^ Adjusted for age, sex, APOE4, education, clinical diagnosis, GDS, VRS, alcohol intake, and dietary pattern.

^b^ Adjusted for covariates in Model 1 plus blood nutritional markers.

### Association between physical activity categories and cognition

Compared to individuals with low PASE scores, those with high PASE scores had higher episodic memory but not higher non-memory cognition (Figure 1 and Table 3). The high PASE score group also had higher total cognition than the low PASE score group. There were no differences in non-memory scores between groups.

**TABLE 3:** Results of multiple linear regression analyses for the associations between PASE categories and cognition.

	Total score		Episodic memory score		Non-memory score	
	**β**	***p***	**β**	***p***	**β**	***p***
**Overall**						
**Model 1** ^a^						
High	0.124	0.032	0.140	0.007	0.067	0.335
Medium	0.075	0.201	0.075	0.152	0.079	0.264
Low	Reference		Reference		Reference	
**Model 2** ^b^						
High	0.128	0.028	0.146	0.005	0.068	0.338
Medium	0.066	0.256	0.067	0.204	0.074	0.302
Low	Reference		Reference		Reference	
**APOE4-negstive**						
**Model 1** ^a^						
High	0.043	0.867	0.117	0.584	−0.179	0.520
Medium	−0.090	0.706	−0.099	0.620	−0.194	0.455
Low	Reference		Reference		Reference	
**Model 2** ^b^						
High	0.107	0.634	0.098	0.637	−0.078	0.775
Medium	−0.208	0.348	−0.188	0.359	−0.366	0.186
Low	Reference		Reference		Reference	
**APOE4-positive**						
**Model 1** ^a^						
High	0.143	0.022	0.140	0.016	0.095	0.206
Medium	0.091	0.146	0.110	0.060	0.098	0.198
Low	Reference		Reference		Reference	
**Model 2** ^b^						
High	0.147	0.018	0.148	0.011	0.097	0.207
Medium	0.096	0.122	0.113	0.054	0.101	0.192
Low	Reference		Reference		Reference	
**Young-old age (65–70 years)**						
**Model 1** ^a^						
High	0.114	0.194	0.077	0.349	0.074	0.518
Medium	0.089	0.335	0.100	0.259	0.182	0.138
Low	Reference		Reference		Reference	
**Model 2** ^b^						
High	0.147	0.107	0.088	0.313	0.108	0.365
Medium	0.068	0.476	0.082	0.368	0.171	0.175
Low	Reference		Reference		Reference	
**Old-old age (> 70 years)**						
**Model 1** ^a^						
High	0.109	0.179	0.202	0.005	0.023	0.805
Medium	0.009	0.917	0.061	0.402	−0.032	0.745
Low	Reference		Reference		Reference	
**Model 2** ^b^						
High	0.099	0.223	0.190	0.008	0.020	0.834
Medium	−0.003	0.970	0.042	0.559	−0.035	0.727
Low	Reference		Reference		Reference	

PASE, physical activity scale for the elderly; APOE4, apolipoprotein ε4; GDS, geriatric depression scale; VRS, vascular risk score.

^a^ Adjusted for age, sex, APOE4, education, clinical diagnosis, GDS, VRS, alcohol intake, and dietary pattern.

^b^ Adjusted for covariates in Model 1 plus blood nutritional markers.

### Moderation effect on the association between physical activity and cognition

There were significant interactions between PASE scores and potential covariables such as age and APOE4 status (Table 4). There were no significant interactions between PASE score and potential covariables such as sex, education, clinical diagnosis, GDS score, VRS, and alcohol intake.

**TABLE 4:** Results of multiple linear regression analyses including PASE × one covariate interaction term, predicting cognition.

	Total score		Episodic memory score		Non-memory score	
	**β**	***p***	**β**	***p***	**β**	***p***
PASE	−0.139	0.439	−0.199	0.212	−0.200	0.356
Age	−0.234	0.010	−0.253	0.002	−0.177	0.102
PASE× Age	0.268	0.157	0.325	0.031	0.308	0.177
PASE	0.081	0.193	0.070	0.212	0.031	0.681
Sex	−0.146	0.122	−0.152	0.071	−0.144	0.204
PASE × Sex	0.061	0.513	0.077	0.356	0.128	0.256
PASE	0.131	0.018	0.101	0.040	0.117	0.078
APOE4	0.054	0.575	−0.064	0.460	0.150	0.198
PASE × APOE4	0.134	0.071	0.154	0.038	0.168	0.157
PASE	0.158	0.140	0.145	0.129	0.155	0.228
Education	0.501	<0.001	0.465	<0.001	0.484	<0.001
PASE × Education	−0.072	0.576	−0.062	0.588	−0.102	0.509
PASE	0.073	0.260	0.036	0.523	0.033	0.670
Clinical diagnosis	−0.548	<0.001	−0.739	<0.001	−0.359	<0.001
PASE × Clinical diagnosis	0.076	0.410	0.147	0.073	0.106	0.345
PASE	0.067	0.443	0.072	0.353	0.045	0.668
GDS	−0.135	0.125	0.023	0.771	−0.208	0.050
PASE × GDS	0.060	0.588	0.043	0.668	0.056	0.677
PASE	0.165	0.022	0.118	0.068	0.132	0.129
VRS	0.138	0.109	0.025	0.745	0.198	0.057
PASE × VRS	−0.121	0.239	−0.037	0.686	−0.103	0.404
PASE	0.101	0.146	0.124	0.046	0.026	0.754
Alcohol intake	0.122	0.187	0.173	0.036	0.015	0.895
PASE× Alcohol intake	0.009	0.925	−0.051	0.560	0.114	0.335

PASE, physical activity scale for the elderly; APOE4, apolipoprotein ε4; GDS, geriatric depression scale; VRS, vascular risk score.

### Association between physical activity and cognition according to age and APOE4 status

Physical Activity Scale for the Elderly scores and episodic memory were positively associated in older adults aged > 70 years and in APOE4-positive individuals (Figure 1 and Tables 3, 4). PASE scores were associated with TS only in APOE4-positive individuals. There were no associations between PASE scores or categories and non-memory scores.

## Discussion

This study of physically capable older adults with no dementia showed that physical activity was associated with higher episodic memory, but not non-memory cognition. Specifically, the beneficial effects of physical activity on episodic memory were apparent in adults aged > 70 years and in APOE4-positive adults.

The observed association between physical activity and episodic memory as the earliest cognitive change in AD (Howieson et al., 1997; Grober et al., 2000; Laakso et al., 2000; Bäckman et al., 2001, 2005; Tromp et al., 2015), is consistent with the results from some previous human studies that demonstrated that physical activity has beneficial effects against AD and cognitive decline. A systematic review and meta-analysis showed that physical activity was associated with lower incidence of AD, with a significant spline trend for AD in dose response meta-analyses (Iso-Markku et al., 2022). The Nurses’ Health Study (Weuve et al., 2004) indicated that physical activity is associated with better cognitive function and less cognitive decline in older women. A population-based cohort study revealed that leisure-time physical activity at least twice per week at midlife was associated with a lower risk for AD and dementia (Rovio et al., 2005). A community-based prospective cohort study of atherosclerosis and cardiovascular disease showed that leisure-time physical activity was associated with lower incidence of dementia and lower cognitive decline (Palta et al., 2019). However, these studies assessed dementia and AD risk in terms of global cognitive performance, rather than AD-related cognition performance. Furthermore, they did not detect the moderation effect of confounding variables on the association between physical activity and episodic memory cognition and did not assess nutritional and blood markers, which may be closely related to physical activity. By contrast, we found that physical activity was associated with episodic memory, i.e., AD related cognition and we investigated moderation of effects of age and APOE4 status on the potential association between physical activity and cognition and detected such an effect.

The mechanism underlying the protective effect of physical activity on AD and related cognition remains unclear. There may be several biological mechanisms linking physical activity to AD and related cognitive decline. Numerous studies have indicated that physical activity regulates several gene transcripts and neurotrophic factors that are related to the preservation of cognitive performance and may enhance brain plasticity (Tong et al., 2001; Berchtold et al., 2002; Cotman and Berchtold, 2002; Rovio et al., 2005; Rockwood and Middleton, 2007). Physical activity may also alleviate amyloid burden in the brain, as shown in a transgenic mouse model of AD (Lazarov et al., 2005). Cholinergic activity increases with physical activity, and the regulation of the cholinergic system by exercise has been implicated in physical activity-induced plasticity (Cotman and Berchtold, 2002). Physical activity has also been suggested to enhance cognitive reserve (Kramer et al., 1999). Together, these findings support neuronal plasticity as a possible mechanism for physical activity-mediated regulation of cognitive performance. However, this mechanism requires verification through further research.

Physical activity was not found to be associated with non-AD related cognition. Physical activity can play an important role in vascular health (Paffenbarger et al., 1993), and cognitive decline due to vascular damage is highly likely to cause non-memory cognitive decline (Viswanathan et al., 2009), which may contradict our findings. However, the protective effect of physical activity on vascular health may be only one of numerous mechanisms (Kramer et al., 1999; Tong et al., 2001; Berchtold et al., 2002; Cotman and Berchtold, 2002; Lazarov et al., 2005; Rovio et al., 2005; Rockwood and Middleton, 2007). Furthermore, there remains a significant association between physical activity and cognitive performance regardless of adjustment for vascular risk factors, which indicates that physical activity protects cognitive performance independently of vascular risk factors.

The beneficial effects of physical activity on AD-related cognition were prominent in old-old adults but not young-old adults in this study, perhaps because the former participants group has had a longer duration of exposure to physical activity. In addition, the effects of physical activity may have been greater in old-old aged adults who were vulnerable to AD and related cognitive decline. Our findings revealed that the association between physical activity and episodic memory cognition was influenced by APOE4 status, with a significant positive association observed between physical activity and episodic memory cognition in individuals with APOE4. These findings are consistent with the findings of the Finnish Geriatric Intervention Study to Prevent Cognitive Impairment and Disability (FINGER) trial (Solomon et al., 2018). The FINGER trial has demonstrated that the implementation of healthy lifestyle changes (positive intervention) has a significantly greater impact on cognition among individuals with APOE4, based on within-group analysis, as opposed to between-group analysis (test of interaction) (Solomon et al., 2018). Our findings could be explained by the potential reversal effect of physical activity on the deleterious actions of APOE4. APOE4 has been associated with blood-brain barrier dysfunction and predicts cognitive decline (Montagne et al., 2020). In addition, the different APOE isoforms can affect the clearance of Aβ, with APOE4 potentially hindering Aβ removal compared to non-APOE4 isoforms (Wildsmith et al., 2013). Therefore, if the detrimental effects of APOE4 on the shared Aβ clearance pathway (Kurz and Perneczky, 2011) are counteracted by the protective effects of physical activity, the benefits of physical activity would be more pronounced in the APOE4-positive group. We demonstrated the potential reversal effect of physical activity on the deleterious actions of APOE4, particularly in older adults with APOE4; notably, the effect of APOE4 on cognition is more pronounced in the older population (Liu et al., 2010).

Our study provides some novel findings regarding physically capable non-demented older adults who underwent clinical assessments including overall physical activity evaluation, laboratory blood tests, nutritional or blood marker tests, and comprehensive cognitive tests with multiple cognitive domains. We also controlled the statistical models for potential confounders to investigate the association between physical activity and AD-related cognition as clearly as possible. Nevertheless, our study had some limitations. First, recall bias may have affected the relationship between physical activity and cognitive performance. Approximately 40% of the participants were diagnosed with MCI, showing recent, rather than remote, memory impairments. This inclusion may raise concerns about the accuracy of self-reports for physical activity. However, the physical activity histories of participants with MCI may not be erroneous, because these self-reports depend mainly on remote memory based on long-established lifestyle rather than recent memory. In addition, there was no interaction between PASE scores and clinical diagnosis states (Table 4), and we obtained similar results after adjustment for the clinical diagnosis as an additional covariate in Model 1. Together, these factors suggest that there are no differences in the relationship between physical activity and cognition with and without a clinical MCI diagnosis. Second, physical activity may be restricted by preexisting brain pathologies because it is a complex motor performance requiring coordination of all body parts, similar to gait (Park et al., 2012). To investigate the precise effect of physical activity on AD-related cognitive domain, we enrolled all participants who were physically capable, i.e., able to perform gait without assistance (UPDRS walking score ≤2). Finally, we did not measure physical activity using objective tools. One pedometer-based study on physical activity (Rabin et al., 2019) assessed the number of steps per day over a short period; however, they did not assess overall physical activity or the various physical activities in which participants engaged. Further investigations using objective measuring instruments that can more fully reflect physical activity are needed to clarify the associations detected in this study.

## Conclusion

Our findings suggest that physical activity had beneficial effects on episodic memory, as an AD-related cognitive domain, in individuals aged > 70 years and in APOE4-positive individuals. Physicians should take age and APOE4 status account into when recommending physical activity to prevent AD-related cognitive decline.

## Data availability statement

The study data are not freely accessible because the Institutional Review Board of the Hallym University Dongtan Sacred Heart Hospital prevents public sharing of such data for privacy reasons. However, the data are available on reasonable request after Institutional Review Board approval. Requests for data access can be submitted to an independent administrative coordinator by e-mail (yoon4645@gmail.com).

## Ethics statement

The studies involving human participants were reviewed and approved by the Institutional review board of the Hallym University Dongtan Sacred Heart Hospital. The patients/participants provided their written informed consent to participate in this study.

## Author contributions

JK conceived and designed the study and served as principal investigator and supervised the study. BL, YC, G-HS, I-GC, HK, JH, DY, and JK were involved in acquisition, or analysis and interpretation of the data and helped to draft the manuscript and were major contributors in writing the manuscript, and critically revising the manuscript for intellectual content. All authors read and approved the final manuscript.
